# Dissolution Reaction and Surface Modification of UICC Amosite in Mimicked Gamble’s Solution: A Step towards Filling the Gap between Asbestos Toxicity and Its Crystal Chemical Features

**DOI:** 10.3390/nano13222933

**Published:** 2023-11-12

**Authors:** Alessandro Pacella, Paolo Ballirano, Maria Cristina Di Carlo, Marzia Fantauzzi, Antonella Rossi, Elisa Nardi, Cecilia Viti, Lorenzo Arrizza, Antonella Campopiano, Annapaola Cannizzaro, Andrea Bloise, Maria Rita Montereali

**Affiliations:** 1 Department of Earth Sciences, Sapienza University of Rome, Piazzale Aldo Moro 5, 00185 Rome, Italy; paolo.ballirano@uniroma1.it (P.B.); mariacristina.dicarlo@uniroma1.it (M.C.D.C.); 2 Rectoral Laboratory Fibres and Inorganic Particulate, Sapienza University of Rome, Piazzale Aldo Moro 5, 00185 Rome, Italy; 3 INSTM Research Unit, Department of Chemical and Geological Sciences, University of Cagliari, 09042 Monserrato, Italy; fantauzzi@unica.it (M.F.); rossi@unica.it (A.R.); 4 Institute for Environmental Protection and Research, ISPRA, Via Vitaliano Brancati 48, 00144 Rome, Italy; elisa.nardi@isprambiente.it; 5 Department of Physical, Earth and Environmental Sciences, University of Siena, Via Laterina 8, 53100 Siena, Italy; cecilia.viti@unisi.it; 6 Microscopy Center, University of L’ Aquila, Via Vetoio, Locality Coppito, 67100 L’Aquila, Italy; lorenzo.arrizza@univaq.it; 7 Department of Medicine, Epidemiology, Occupational and Environmental Hygiene, National Institute for Insurance against Accidents at Work (INAIL), Via Fontana Candida 1, 00078 Rome, Italy; a.campopiano@inail.it (A.C.); a.cannizzaro@inail.it (A.C.); 8 Department of Biology, Ecology and Earth Sciences, University of Calabria, V. P. Bucci, 87036 Arcavacata di Rende, Italy; 9 Italian National Agency for New Technologies, ENEA, Casaccia Research Centre, Via Anguillarese 301, 00123 Rome, Italy; mariarita.montereali@enea.it

**Keywords:** asbestos, UICC amosite, fibre dissolution, Gamble’s solution

## Abstract

This study focuses on the dissolution process and surface characterization of amosite fibres following interaction with a mimicked Gamble’s solution at a pH of 4.5 and T = 37 °C, up to 720 h. To achieve this, a multi-analytical approach was adopted, and the results were compared to those previously obtained on a sample of asbestos tremolite and UICC crocidolite, which were investigated under the same experimental conditions. Combining surface chemical data obtained by XPS with cation release quantified by ICP-OES, an incongruent behaviour of the fibre dissolution was highlighted for amosite fibres, similarly to asbestos tremolite and UICC crocidolite. In particular, a preferential release of Mg and Ca from the amphibole structure was observed, in agreement with their Madelung site energies. Notably, no Fe release from amosite fibres was detected in our experimental conditions (pH of 4.5 and atmospheric pO_2_), despite the occurrence of Fe(II) at the *M*(4) site of the amphibole structure, where cations are expected to be rapidly leached out during mineral dissolution. Moreover, the oxidation of both the Fe centres initially present on the fibre surface and those promoted from the bulk, because of the erosion of the outmost layers, was observed. Since biodurability (i.e., the resistance to dissolution) is one of the most important toxicity parameters, the knowledge of the surface alteration of asbestos possibly occurring in vivo may help to understand the mechanisms at the basis of its long-term toxicity.

## 1. Introduction

The term asbestos refers to a group including five fibrous amphiboles (amosite, i.e., the fibrous variety of cummingtonite-grunerite; tremolite; actinolite; crocidolite, i.e., the fibrous variety of riebeckite; and anthophyllite) and one fibrous serpentine mineral (chrysotile) [1]. Since the industrial age, asbestos has been intensively used in many products, mostly including building materials, friction pads, and heat-resistant fabrics, due to its physical and chemical properties, such as high mechanical strength, thermal insulation, and resistance to high temperature [2]. The global asbestos industry experienced rapid expansion during the 1960s and 1970s [3], and approximately 210 million tons of asbestos fibres were extracted worldwide between 1900 and 2015 [4]. However, after World War II, an increasing number of scientific studies revealed that the inhalation of asbestos can cause malignant mesothelioma, lung diseases like carcinoma, asbestosis, and others [5]. Accordingly, in 1989, the International Agency for Research on Cancer (IARC) classified asbestos as Group 1 “substances carcinogenic to humans” [6]. Today, asbestos use is banned in more than 50 countries (International Ban Asbestos Secretariat, 2019) [7]. 

Today, the toxicity of inhaled asbestos is considered the result of a complex multistep process governed by the interplay of all the physical/chemical and structural characteristics of fibres, including morphology, chemical composition, surface reactivity, and biodurability/biopersistence [8,9,10,11,12,13,14,15,16]. Among these factors, Fenton active iron ions exposed on the fibre surface are considered to play a primary role by yielding HO^•^ radicals, which have a high potency to damage DNA, proteins, and lipids, and hence to induce carcinogenicity [17,18,19,20]. Because of this complexity, there are still open issues and disputes about the molecular mechanism/s at the basis of the toxicity and carcinogenicity of mineral fibres [21].

Recently, surface reactivity studies on fibrous amphiboles (crocidolite and tremolite) carried out by some of the current authors highlighted that the HO^•^ production is related to specific surface Fe sites rather than the total Fe content of the minerals [22,23,24]. Moreover, it was established that due to leaching under physiological conditions, the surface modifications of the fibres may change the radical yield of amphibole asbestos depending on fibre crystal chemistry, surface area, and leaching solution [22,23].

Hence, the knowledge of the fibre alteration occurring in simulated biological fluids is the prerequisite step to understand how fibres may react with their biological surrounding. Notably, this piece of information may help to shed new light on the mechanisms of asbestos-induced toxicity. 

This work focuses on the surface modifications of amosite fibres during incubation in a mimicked Gamble’s solution (MGS) at pH 4.5, up to one month. Leached fibres were studied by using a multi-analytical approach: field-emission scanning electron microscopy (FE-SEM) was used to characterize the fibre morphology and inductively coupled plasma optical emission spectrometry (ICP-OES) was employed to measure the cation release into the leaching solution. The analytical approach based on X-ray photoelectron spectroscopy (XPS), exploited for characterizing the other asbestos fibres, was here adapted to monitoring possible changes of surface chemistry, and includes the Fe speciation of the amosite. Powder X-ray diffraction (PXRD) and high-resolution transmission electron microscopy (HR-TEM) were used to observe possible nanostructural modification of the fibres. The results are discussed comparing them with those obtained on asbestos tremolite and crocidolite previously investigated under the same experimental conditions [25]. 

## 2. Materials and Methods

### 2.1. Materials

A sample of amosite (fibrous grunerite) standard sample from Penge mine (South Africa), supplied by the Union International for Cancer Control (UICC), was investigated in this work. The detailed crystal chemical and structural characterization of the sample is reported in Ballirano et al. [26]. The corresponding empirical formula is: ^A^(Na_0.02_)_Σ0.02_^B^(Fe^2+^_1.54_Mn_0.29_Na_0.10_Ca_0.07_)_Σ2.00_^C^(Fe^2+^_2.92_Mg_1.93_Fe^3+^_0.15_)_Σ5.00_^T^(Si_7.93_Al_0.07_)_Σ8.00_O_22.00_^W^(OH_2.00_)_Σ2.00_.

Rietveld refinement results evidenced that Fe^2+^ is allocated in the octahedral layer following the site preferences *M*(1) ≈ *M*(3) > *M*(2), whereas Mg is preferentially ordered at *M*(2). A quantitative phase analysis indicated about 10 wt.% of accessory phases including quartz (ca. 4 wt.%), ankerite (ca. 1 wt.%), and traces of stilpnomelane and biotite/annite. The surface area of the samples, measured by nitrogen physisorption (BET), resulted to be 4.5 m^2^ × g^−1^.

### 2.2. Dissolution in MGS 

Dissolution experiments were carried out under static conditions in the same experimental conditions used for UICC crocidolite and fibrous tremolite [25]. Briefly, samples were incubated (20 mg) in MGS (40 mL) at pH 4.5 and kept at 37 °C for up to 1 month with gentle shaking. Aliquots of the suspension were filtered on nitrocellulose filter membranes (porosity of 0.22 µm) and analysed by ICP-OES using a Perkin–Elmer Optima 2000 DV ICP-OES spectrometer (Perkin–Elmer, Norwalk, CT, USA). To remove any residues of the solution, the fibres deposited on filters were rinsed with ultrapure deionized water and then stored under argon prior to the SEM, TEM, XRPD, and XPS investigations. 

### 2.3. FE-SEM Investigation

SEM images were acquired by using FESEM Thermo Fischer Apreo 2 S LoVac (Brno-Černovice, Czech Republic). Samples were mounted on a stub with conductive carbon tape, and a 5 nm of chromium film was deposited on the sample surface to make it conductive during measurements.

### 2.4. HR-TEM Investigation

A TEM analysis was performed using a JEOL JEM-2010 (Tokyo, Japan) microscope operating at 200 kV, equipped with a LaB6 source, an Energy Dispersive System (EDS) (Oxford ISIS, Oxford, UK) for microanalysis, and an Olympus Tengra CCD camera (2 k × 2 k × 14 bit) for image acquisition. Aliquots of the samples (pristine amosite and 1 month in MGS incubated amosite) were pipetted on 200-mesh Cu grids supported with holey carbon film, then carbon-coated to increase conductivity. 

### 2.5. Powder X-ray Diffraction (PXRD)

X-ray powder diffraction data were collected on a D8 Advance (Bruker AXS, Karlsruhe, Germany) running in θ/θ transmission mode using a capillary as a sample holder. The sample incubated for 1 month in MGS (A-720 h) was loaded in a 0.5 mm diameter borosilicate glass capillary. The instrument is equipped with an incident beam focussing graded multilayer Göbel mirror and a PSD VÅntec-1. The diffraction pattern was measured in step-scan mode, using CuKα, in the 6–145° 2θ angular range, 0.022° 2θ step size, and 20 s counting time. Data were evaluated by the Rietveld method using Topas V6 (Bruker AXS, Billerica, MA, USA, 2016), which uses the fundamental parameters approach (FPA) [27] for describing the peak shape. Preliminary scrutiny of the pattern indicated the persistence of the same phases at the minor/trace level reported in Ballirano et al. [26], and the same computational procedure was applied. Quartz, ankerite, and garnet were included in the Rietveld refinement. Differently, the main reflections of stilpnomelane and biotite/annite were approximated by two peaks not related to any structure, whose position, intensity, and breadth were also refined following the same procedure described by Pacella et al. [25]. Only scale factors, cell parameters, and peak shapes were refined for quartz, ankerite, and garnet. The structure of amosite was refined keeping all displacement parameters fixed to reference data [28], and no restraints on bond distances and angles were imposed. Site scattering (s.s.) at *M*(1), *M*(2), *M*(3), and *M*(4) was optimized. Neither split M(4′) site nor A-type sites were observed in agreement with Ballirano et al. [26]. The refinement was performed using the normalized symmetrized spherical harmonics functions, reported by Järvinen [29], for describing the anisotropic peak broadening of the diffraction pattern. Absorption correction was performed using the equation of Sabine et al. [30] for a cylindrical sample, and the presence of preferred orientation was modelled using normalized symmetrized spherical harmonics functions (fourth order, eight refinable parameters) following the approach of Ballirano [31]. The results of the quantitative phase analysis (QPA) are reported in Table 1. Statistical indicators of the refinement and cell parameters of A-720 h are listed in Table S1, whereas a magnified view (5–80° 2θ) of the Rietveld plots is shown in Figure S1. The CIF file of A-720 h is deposited as supporting material at the journal’s site.

### 2.6. XPS Investigation

An XPS investigation was carried out by using a Theta Probe X-ray photoelectron spectrometer (Thermo Fisher Scientific, Waltham MA, USA) equipped with a flood gun neutralizer for charge compensation. All spectra were acquired using Al Kα1,2 source (hν = 1486.6 eV) and a 400 μm spot size. The analyser operated in the fixed analyser transmission (FAT) mode, with pass energy (PE) set at 200 eV and at 100 eV for the survey spectra and for the high-resolution spectra, respectively. The linearity of the binding energy scale was checked by periodic calibration following ISO 15472:2010 [32]. The binding energy scale was referred to the signal at 285.0 eV of adventitious aliphatic carbon. More details on the curve fitting of the spectra are reported in our previous works [33]. The surface fibre composition was reported in atomic percentages, and it was calculated correcting the peak area for Scofield’s photoionization cross-sections [34], the asymmetry function [35], the transmission function correction, and the attenuation length [33]. Data are reported as the average values of three different analysed areas, with standard deviation in brackets.

## 3. Results 

### 3.1. Morphological Investigation

**Figure 1:**
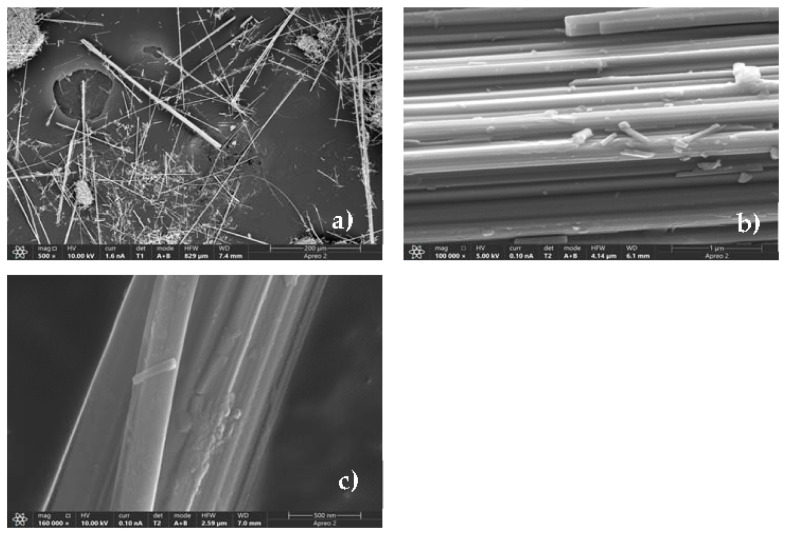
FE-SEM images of amosite fibres: (**a**) pristine amosite showing straight and rigid morphology arranged in bundles; (**b**) high magnification image showing pristine fibrils with nanometric diameter (ca. 150–300 nm); (**c**) high magnification image of fibres after incubation in MGS for up to 1 month showing an irregular surface due to dissolution process.

The FE-SEM images show that amosite fibres, similarly to crocidolite fibres [25], are straight and rigid, and are arranged in bundles of variable dimensions of ca. 1–5 μm × 20–1000 μm (diameter × length) with split ends (Figure 1a). Images at high magnification (Figure 1b) show single fibrils of nanometric diameter (ca. 150–300 nm) with a partially irregular surface, indicating the possible occurrence of an amorphous layer due to weathering processes [25]. Moreover, after immersion in MGS for up to 1 month (sample A-720 h), in rare cases, some effects of the dissolution are evident on the fibres, their surface being more lobate and irregular with respect to that of the starting material (Figure 1c). 

**Figure 2:**
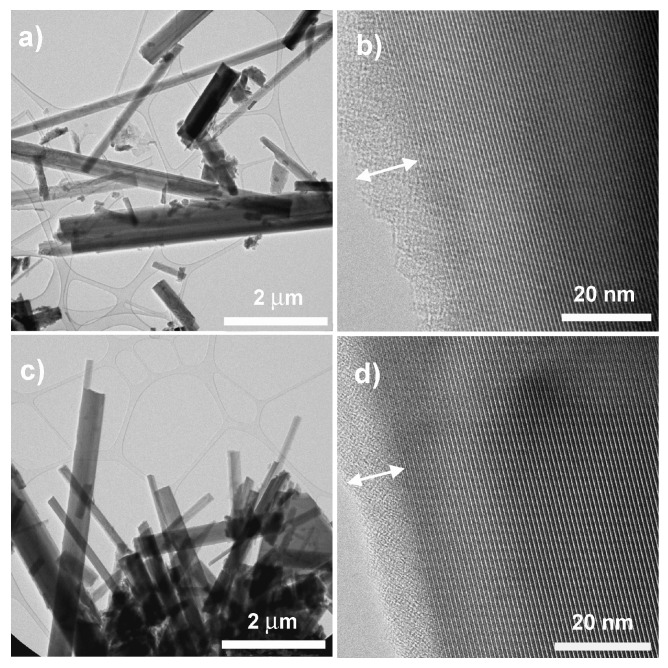
Bright-field TEM images of pristine amosite (**a**,**b**) and amosite after 1 month of incubation (**c**,**d**) at variable magnification. Lattice fringes in (**b**,**d**) correspond to (020) with d-spacings of 9.05–9.20 Å. Double-arrowed white lines highlight the thin amorphous film surrounding both pristine and treated amosite fibres.

The TEM observations reveal similar nanostructures for pristine and A-720 h samples. In both samples, fibre size and aspect ratio are highly variable, with a maximum diameter of 500–600 nm (e.g., Figure 2a,c, for pristine and A-720 h samples, respectively). High-magnification images reveal the constant occurrence of an ultrathin amorphous film, approximately 10–30 nm wide, surrounding the amosite fibres (Figure 2b,d). The amorphous film also occurs at fibre terminations. The only remarkable difference between pristine amosite and A-720 h treated fibres is an average increase in the amorphous film thickness at fibre termination in the treated sample (up to 25 nm), where rare irregular, wavy lattice fringes may occur, and suggesting possible precipitation of smectite-like layers. 

### 3.2. Structural Analysis

The cell parameters and volume of the A-720 h sample are very close to those of the pristine one (Table S1). The QPA results testify the complete dissolution of ankerite observed in the pristine sample. Moreover, the presence of an extra reflection at ca. 18° 2θ, already observed for other amphiboles (UICC crocidolite and tremolite: [25]) upon dissolution experiments in MGS at acidic conditions, was assigned to newly precipitated hydrated sulphates. Relevant bond distances of A-720 h are reported in Table S2, where they are compared to those of the pristine sample [26]. The differences are marginal and testify the absence of significant structural variations at the bulk level. The same applies to the site scattering (s.s.) of the cation sites (Table S3) and the site partition at the B and C sites (Table S4). Cation partition was calculated assuming that all Mn is allocated at *M*(4). 

Comparison with the pristine sample possibly indicates only a minor oxidation of Fe^2+^ at C sites. The <<*M*(1), *M*(2), *M*(3)–O>> is of 2.111 Å as compared to 2.114 Å of the pristine sample [26]. The C group sites preference of Fe^2+^ follow the same *M*(1) ≈ *M*(3) > *M*(2), with Mg preferentially ordered at *M*(2), of the pristine sample, in agreement with the reference data of Hirschmann et al. [36]. An indirect Fe^2+^/Fe^3+^ partition was performed by comparing the <r*_M_*> mean cationic radii calculated from both the refined <*M*(1,2,3)–O> bond distances and from the proposed site partition (Table S4). The results confirm the unusual preferential allocation of Fe^3+^ at *M*(1) (0.15 apfu) and *M*(3) (0.09) observed in the pristine sample, plus minor Fe^3+^ at *M*(2) (0.03 apfu) for a total of 0.27 Fe^3+^ apfu to be compared to 0.15 Fe^3+^ apfu of the untreated sample.

### 3.3. Dissolution Process

**Figure 3:**
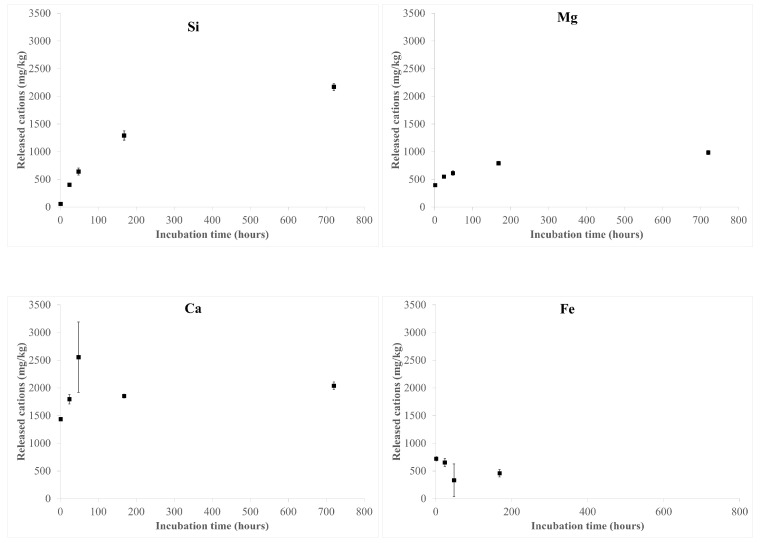
Released cations from the UICC amosite sample during incubation in MGS at pH 4.5 for up to 1 month (720 h).

The results of the ICP-OES analyses after fibre incubation in the MGS are shown in Figure 3 and Table S5. Si and Mg release shows a parallel trend, both increasing with incubation time, from 57(1) mg/kg up to 2169(62), and from 394(3) mg/kg up to 985(36) mg/kg, respectively (Figure 3). 

Ca and Fe release mainly occurs in the first stages of dissolution (48 and 24 h of incubation, respectively). The observed release of Fe and Ca may be attributed to the dissolution of ankerite [calcium, iron carbonate: CaFe(CO_3_)_2_] in the hand sample, in agreement with the QPA results showing the absence of this accessory phase in the sample incubated for up to 1 month in MGS (Table 1). It must be pointed out that the concentration of released Ca is higher than that expected from the dissolution of only ankerite: considering the dissolution of ca. 0.76 wt.% of ankerite, the concentration of Ca and Fe should be roughly 1500 and 1200 mg/kg, respectively. Thus, due to the occurrence of the small content of Ca in the amosite structure, Ca leaching from the fibres cannot be ruled out. Moreover, the small amount of Fe detected in the solution (up to 720 mg/kg) indicates the possible formation of Fe-bearing secondary phases (hydrated sulphates), as revealed by PXRD data reported in Table 1). This hypothesis is further supported by the absence of Fe in the solution for longer incubation times (1 month). 

### 3.4. Surface Chemistry

The XPS survey spectra of the amosite samples after suspension in MGS are reported in Figure S2 and show the presence of Si, O, Fe, Mg, Ca, and Na, together with C, due to the presence of the adventitious carbon caused by the contact with the laboratory atmosphere and aqueous solutions. Specifically, no S and Cl signals that might be due to the incubating solution were found on the fibre surface. The binding energy of the principal photoelectron lines are shown in Table S6. The high-resolution spectra were processed to gain data on the chemical state of the elements. 

Si 2p peaks were fitted with a doublet due to spin orbit coupling. The energy separation between the 2p_3/2_ and 2p_1/2_ components and their area ratio were constrained to 0.8 and 2:1, respectively. The binding energy of Si 2p_3/2_ was found to be in the range 102.5 (0.1)–102.7 (0.1) for the considered samples (Table S6) and agrees with that reported in our previous investigations [33]. The oxygen O 1s peak resulted to be multicomponent with a signal due to oxygen in oxides in the range 530.0–530.2 eV; the component due to bridging oxygen is at 532.2 eV, and the components assigned to non-bridging oxygen in silicates and -OH are found at about 531.2 eV [33]. The quantitative surface composition of both pristine and MGS-incubated fibres is reported in Table 2. Compared to the bulk composition, an enrichment in Mg and Si with respect to Fe is evident on the surface of pristine fibres (Table 2), likely due to weathering processes, as we already observed for UICC crocidolite fibres [25]. It must be pointed out that, owing to their very low amount in the hand sample, the contribution of accessory phases to the XPS measurements may be considered negligible. 

The Fe 2p_3/2_ high-resolution spectra of the samples are shown in Figure S3. Following Fantauzzi et al. [33], the signals were resolved in three components assigned to: (i) Fe(II) bound to oxygen with its satellite, at about 709.0 eV; (ii) Fe(III) bound to oxygen found at 710.6 eV; and (iii) FeOOH at 711.7 eV. The percentage of each component is shown in Table 3. Significant variations of the Fe components were observed only in the first hour of sample incubation in MGS. In particular, the intensity of the Fe(II)-O signal decreases from ca. 70% of the total peak area in the pristine sample down to ca. 60%, at the expense of the Fe(III)-O signal, which increases from ca. 3% up to ca. 18%. Moreover, the signals remain almost constant up to the end of the experiment.

## 4. Discussion

Cation release as a function of the sample incubation time is reported in Figure 3 and Table S5. It must be pointed out that the observed release of Fe and (at least a fraction of) Ca was ascribed to the dissolution of a small amount of ankerite, a carbonate mineral soluble at acidic pH, occurring in the hand sample (Table 1).

**Figure 4:**
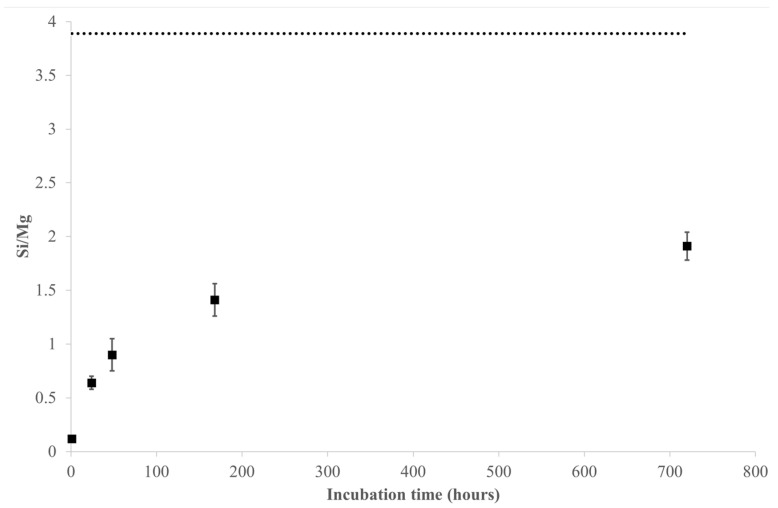
Dissolution of amosite fibres in MGS at pH 4.5 in the range 0–720 h: Si/Mg ratio (as nmol/mg) in the solution at each sampling time as compared to that arising from XPS analysis of the pristine sample. Dotted line shows the Si/Mg ratio on the fibre surface, retrieved from XPS analysis.

Comparing the Si/Mg ratio of the leached cations (based on nanomoles) at the various sampling times with that retrieved from the surface chemical analysis (Figure 4), a preferential Mg release from the amosite fibres is evident, especially in the first 48 h, as already observed for the dissolution of asbestos tremolite and UICC crocidolite samples [25]. Accordingly, the incongruent behaviour of the dissolution process was also highlighted by the XPS results (Table 2 and Table 4) showing a depletion of Mg and Ca on the surface of the leached fibres, in agreement with their Madelung site energies in the amphibole structure [37,38]. 

Notably, the selective removal of cations from the amphibole structure, leading to surface amorphization, was highlighted by Germine et al. [39] on fibrous tremolite samples following in vivo alteration. Later, Germine et al. [40] found that the neo-formed surface layer is not completely amorphous but is composed of sub-nanometer silica-rich particles with the potential to penetrate deeply into DNA interiors. The Fe enrichment on the fibre surface (Table 4) indicates no Fe leaching from the amosite fibres in the adopted experimental conditions (pH of 4.5 and atmospheric pO_2_), despite significant Fe(II) occurrence at the *M*(4) site [26], where cations are expected to be rapidly leached out during amphibole dissolution on the basis of its Madelung site energy [37,38,41]. Moreover, the Na enrichment on the sample surface during incubation is likely due its adsorption on the fibre surface from the MGS solution (Table 4).

Concerning Fe speciation, pristine fibres show a more oxidized surface with respect to the bulk (Fe(II)/Fe_tot_ ratios are 97% for the bulk and 71% for the surface), with Fe(III) mainly present as FeOOH (26% of the total surface Fe content). For the MGS incubated samples (Table 3), the intensity of the Fe(II)-O component decreases in the first hour of incubation, from about 71% to ca. 59% of the total Fe, and is coupled with a parallel increase in the Fe(III)-O component (from ca. 3% to ca. 15% of the total Fe). Moreover, the increase in the Fe(III)-O component on the surface is in agreement with the increased oxygen content observed in the same interval of time (Table 4). The depletion of Fe(II) centres on the surface indicates that for amosite fibres the Fe oxidation is faster than the fibre dissolution in the first hour of incubation, similarly to what was observed for samples of fibrous tremolite [25]. However, for amosite fibres, the Fe(II) reduction is less marked, being only ca. 17% of the total surface Fe(II), whereas for tremolite it is much more pronounced [ca. 62% of the total surface Fe(II) present in the pristine sample is oxidized in the same interval of time]. Moreover, in the asbestos tremolite sample, the oxidation converted Fe(II) into FeOOH (Table 3), very likely located in the outer part of the sample surface [33]. This might be attributed to the slow dissolution rate of the investigated fibres, affected by both their crystal chemical features and low surface area (ca. 2.7 m^2^ × g^−1^ for Maryland tremolite, see Pacella et al. [25]). Notably, the observed trend is opposite to that of UICC crocidolite, which shows an increase in the Fe(II) component coupled with a decrease in the FeOOH component (Table 3). This is a consequence of the fast fibre dissolution that removes the outer layer and promotes the exposure of new Fe sites from the bulk, especially in the form of Fe(II) of which the bulk is enriched with respect to the surface. Considering that for amosite fibres the FeOOH component keeps constant with incubation time, the moderate conversion of Fe(II)-O to Fe(III)-O observed in the first hour of incubation (Table 3) may be interpreted as the result of both the oxidation of the Fe centres initially present on the fibre surface and those emerging from the bulk following mineral dissolution. Those interpretations are supported by the Rietveld structural analysis that indicates a minor oxidation of Fe(II) of the bulk. Notably, for amosite (ca. 4.5 m^2^ × g^−1^), the lower surface area with respect to that of crocidolite (ca. 8.7 m^2^ × g^−1^) likely hinders a sustained occurrence of Fe(II) centres on the fibre surface (Table 3). This agrees with previous results obtained on two fibrous tremolite samples with largely different surface areas revealing that both the processes of dissolution and surface modification are slower for the sample with the lowest surface area [23]. Moreover, starting from 24 h of sample incubation, an equilibrium between Fe oxidation rate and fibre dissolution kinetics is established (Table 3). To make a comparison of the biodurability among these amphibole asbestos samples, the dissolution rate of amosite fibres normalized to their surface area was quantified, according to Pacella et al. [25], using the Si release in the unsaturated region (0–48 h). The value obtained was dSi/dt = 0.002 µmol × h^−1^ × m^−2^ (R^2^ = 0.99), in between that retrieved from UICC crocidolite and asbestos tremolite (dSi/dt = 0.007 µmol × h^−1^ × m^−2^ and 0.0004 µmol × h^−1^ × m^−2^, respectively). This result unequivocally confirms previous findings postulating: (i) a quicker dissolution for Fe-rich silicates with respect to their isostructural, iron-free analogues [37,42]; (ii) asbestos tremolite durability is among the highest compared to any other amphibole asbestos [43,44,45,46,47,48].

## 5. Conclusions

In this work, the dissolution process in MGS at acidic pH (ca. 4.5), and the following chemical and structural surface alterations of UICC amosite fibres, were investigated by a well-tested multi-analytical approach. The results obtained were compared with those previously acquired, under the same experimental conditions, on asbestos tremolite and UICC crocidolite samples. 

Despite a similar dissolution process (i.e., incongruent dissolution with preferential release of Mg and Ca leading to surface amorphization, and absence of Fe release), differences in biodurability among the samples were highlighted. In particular, the release of Si based on an equivalent surface area showed that amosite fibres have an intermediate biodurability between that of crocidolite (lowest biodurability) and asbestos tremolite (highest biodurability). Moreover, for both amosite and asbestos tremolite samples, the transformation of bulk Fe(II) centres to surface ions is overwhelmed by the Fe oxidation rate in the first stages of dissolution. Accordingly, a depletion of surface Fe(II) is observed for the leached samples with respect to the pristine ones.

The results of our group showed that the alteration of the fibre surface following dissolution may induce the occurrence of under-coordinated Fe ions, which primarily contribute to the overall fibre reactivity. On this basis, the fibre dissolution kinetics can drive the extent of surface alteration that in turn modulates the fibre chemical reactivity and, possibly, its ability to interact with the biological environment in vivo (i.e., radical generation by Fenton reaction, protein adsorption, etc.). Since biodurability is assumed to be a relevant toxicity parameter, the knowledge of the surface alteration fibres possibly occurring in lung fluids is of primary importance to assess the mechanisms underlying the observed long-term toxicity of asbestos. To provide a much more detailed picture of the possible surface modifications occurring in vivo, other works focusing on the treatment of fibres with real fluids present in the lungs are in progress.

## Figures and Tables

**Table 1:** QPA of the UICC amosite samples. Data of pristine samples taken from Ballirano et al. [26].

Phases	Pristine	A-720 h
Amosite	94.87(8)	95.74(8)
Quartz	3.79(5)	3.86(7)
Ankerite	0.76(4)	
Garnet	0.58(4)	0.40(4)
Stilpnomelane	traces	traces
Biotite/annite	traces	traces
Hydrated sulphates	-	traces

**Table 2:** Surface quantitative composition (at%) of both pristine and amosite samples after incubation in MGS. Average values and standard deviation (in parentheses) over three measurements are reported. The composition of the bulk obtained by chemical analysis is reported for comparison.

Sample	O	Si	Mg	Fe	Na	Ca
Bulk	62.1	20.5	5.0	11.9	0.3	0.2
Pristine	57.8 (0.4)	28.4 (0.5)	7.3 (0.2)	5.9 (0.3)	n.d.	0.6 (0.2)
A-1 h	62.1 (0.7)	24.7 (0.8)	6.0 (0.8)	4.9 (0.1)	2.0 (0.3)	0.3 (0.1)
A-24 h	62.4 (0.6)	24.8 (0.7)	5.5 (0.2)	5.35 (0.05)	1.8 (0.3)	0.15 (0.03)
A-48 h	63.1 (0.5)	24.5 (0.2)	4.6 (0.4)	5.9 (0.1)	1.9 (0.4)	n.d.
A-168 h	63.6 (0.6)	23.4 (0.4)	5.3 (0.3)	6.1 (0.2)	1.5 (0.2)	n.d.
A-720 h	63.7 (0.2)	24.4 (0.7)	4.5 (0.4)	6.2 (0.2)	1.1 (0.1)	n.d.

**Table 3:** Relative intensities of Fe 2p_3/2_ components (area%) in UICC amosite samples (A). Results of Pacella et al. [25] obtained for UICC crocidolite (C) and asbestos tremolite (T) investigated under the same experimental conditions are reported for comparison.

	A: UICC Amosite (This Work)				B: UICC Crocidolite (Pacella et al. [25])				C: Tremolite (Pacella et al. [25])		
Sample	Fe(II)-O	Fe(III)-O	Fe-OOH	Sample	Fe(II)-O	Fe(III)-O	Fe-OOH	Sample	Fe(II)-O	Fe(III)-O	Fe-OOH
Pristine	71(3)	3(1)	26(4)	Pristine	29	10	61	Pristine	21(1)	19(1)	60(1)
A-1 h	59(3)	18(4)	23(2)	T-1 h	11(2)	5(1)	84(3)	C-1 h	24(1)	20.8(0.2)	55.3(0.2)
A-24 h	65(1)	12(1)	23(1)	T-24 h	13(1)	2(1)	85(1)	C-24 h	25(1)	23(1)	53(2)
A-48 h	59(3)	15(2)	26(4)	T-48 h	19(1)	3(1)	78(2)	C-48 h	30.5(0.2)	20.6(0.9)	49(1)
A-168 h	56(1)	18.0(0.1)	26(1)	T-168 h	13(1)	10(2)	77(3)	C-168 h	28.9(0.1)	23(3)	48(3)
A-720 h	59(5)	15(3)	26(2)	T-720 h	15(2)	8(2)	77(2)	C-720 h	29(2)	18(1)	53(1)

**Table 4:** Silicon/cation ratios on the surface of the amosite fibres as a function of the incubation time.

Sample	Si/Mg	Si/Fe	Si/O
Pristine	3.9(0.2)	4.8(0.3)	0.49(0.01)
A-1 h	4.1(0.7)	5.0(0.3)	0.40(0.02)
A-24 h	4.5(0.3)	4.6(0.2)	0.40(0.02)
A-48 h	5.3(0.5)	4.2(0.1)	0.39(0.01)
A-168 h	4.4(0.3)	3.8(0.2)	0.37(0.01)
A-720 h	5.4(0.6)	3.9(0.2)	0.38(0.01)

## Data Availability

The data presented in this study are available on request from the corresponding author.

## Supplementary Materials

The following supporting information can be downloaded at: https://www.mdpi.com/article/10.3390/nano13222933/s1, Figure S1. Magnified view (5–80° 2θ) of the Rietveld plots of the refinement of A-720 h. Continuous blue line: experimental data; continuous red line: calculated pattern; continuous grey line: difference pattern. Vertical blue lines: single peak of annite/biotite (ca. 7.25° 2θ), stilpnomelane (ca. 8.8° 2θ), and hydrated sulphates (ca. 18° 2θ) (see text for explanation). Vertical short bars: position of calculated Bragg reflections of (from above to below): garnet, quartz, and amosite; Figure S2. Survey spectra of the investigated UICC amosite samples. X-ray source: Al Kα; Figure S3. Fe 2p_3/2_ peaks of the UICC amosite samples pristine and incubated in mimicked Gamble’s solution for 1 h and 720 h; Table S1. Cell parameters and volume of UICC amosite fibres and agreement factors (as defined in the work of Young [28]) of the Rietveld refinements. Data of pristine samples taken from Ballirano et al. [26]; Table S2. Relevant bond distances (in Å) of UICC amosite fibres. * Calculated as in table 7 in the work of Hawthorne and Oberti [49]. Data of pristine samples taken from Ballirano et al. [26]; Table S3. Site scattering (*s.s.*) at A, B, and C sites from Rietveld refinement. Data of pristine samples taken from Ballirano et al. [26]; Table S4. Site partition at B and C sites from Rietveld refinement. Data of pristine samples taken from Ballirano et al. [26]; Table S5. Results of ICP-OES analyses of the UICC amosite sample after incubation in the mimicked Gamble’s solution at pH 4.5 up to 720 h. Standard deviations (in parentheses) were calculated over three independent measurements; Table S6. Binding energy values (eV) of the main photoelectron lines in UICC amosite samples. Average values and standard deviation (in parentheses) over three measurements carried out on different areas of the same sample.

## References

[1] Skinner H.C.W. (2003). Mineralogy of Asbestos Minerals. Indoor Built Environ..

[2] Kim S.-Y., Kim Y.-C., Kim Y., Hong W.-H. (2016). Predicting the Mortality from Asbestos-Related Diseases Based on the Amount of Asbestos Used and the Effects of Slate Buildings in Korea. Sci. Total Environ..

[3] Iwaszko J. (2019). Making Asbestos-Cement Products Safe Using Heat Treatment. Case Stud. Constr. Mater..

[4] Spasiano D., Pirozzi F. (2017). Treatments of Asbestos Containing Wastes. J. Environ. Manag..

[5] Gualtieri A.F. (2021). Bridging the Gap between Toxicity and Carcinogenicity of Mineral Fibres by Connecting the Fibre Crystal-Chemical and Physical Parameters to the Key Characteristics of Cancer. Curr. Res. Toxicol..

[6] IARC Working Group on the Evaluation of Carcinogenic Risks to Humans. Arsenic, Metals, Fibres and Dusts. Proceedings of the IARC Monographs on the Evaluation of Carcinogenic Risks to Humans.

[7] International Ban Asbestos Secretariat. http://ibasecretariat.org/alpha_ban_list.php.

[8] Fubini B., Mollo L. (1995). Role of Iron in the Reactivity of Mineral Fibers. Toxicol. Lett..

[9] Donaldson K., Murphy F.A., Duffin R., Poland C.A. (2010). Asbestos, Carbon Nanotubes and the Pleural Mesothelium: A Review of the Hypothesis Regarding the Role of Long Fibre Retention in the Parietal Pleura, Inflammation and Mesothelioma. Part. Fibre Toxicol..

[10] Bernstein D., Dunnigan J., Hesterberg T., Brown R., Velasco J.A.L., Barrera R., Hoskins J., Gibbs A. (2013). Health Risk of Chrysotile Revisited. Crit. Rev. Toxicol..

[11] Bonneau L., Malard C., Pezerat H. (1986). Studies on surface properties of asbestos. 2. Role of dimensional characteristics and surface properties of mineral fibers in the induction of pleural tumors. Environ. Res..

[12] Bonneau L., Suquet H., Malard C., Pezerat H. (1986). Studies on surface properties of asbestos. 1. Active sites on surface of chrysotile and amphiboles. Environ. Res..

[13] Gilmour P.S., Brown D.M., Beswik P.H., Macnee W., Rahman I., Donaldson K. (1997). Free radical activity of industrial fibers: Role of iron in oxidative stress and activation of transcription factors. Environ. Health Perspect..

[14] Fubini B., Kane A.B., Boffetta P., Saracci R., Wilbourn J. (1996). Physico-chemical and cell free assays to evaluate the potential carcinogenicity of fibres. Mechanisms of Fibre Carcinogenesis.

[15] Fubini B., Otero Arean C. (1999). Chemical aspects of the toxicity of inhaled mineral dusts. Chem. Soc. Rev..

[16] Van Oss C.J., Naim J.O., Costanzo P.M., Giese Jr R.F., Wu W., Sorling A.F. (1999). Impact of different asbestos species and other mineral particles on pulmonary pathogenesis: Clay. Clay Min..

[17] Hardy J.A., Aust A.E. (1995). Iron in Asbestos Chemistry and Carcinogenicity. Chem. Rev..

[18] Fenoglio I., Prandi L., Tomatis M., Fubini B. (2001). Free Radical Generation in the Toxicity of Inhaled Mineral Particles: The Role of Iron Speciation at the Surface of Asbestos and Silica. Redox Rep..

[19] Kamp D.W. (2009). Asbestos-induced lung diseases: An update. Transl. Res..

[20] Liu G., Cheresh P., Kamp D.W. (2013). Molecular Basis of Asbestos-Induced Lung Disease. Annu. Rev. Pathol. Mech. Dis..

[21] Gualtieri A.F., Gualtieri A.F. (2017). Introduction. Mineral Fibres: Crystal Chemistry, Chemical-Physical Properties, Biological Interaction and Toxicity.

[22] Andreozzi G.B., Pacella A., Corazzari I., Tomatis M., Turci F. (2017). Surface Reactivity of Amphibole Asbestos: A Comparison between Crocidolite and Tremolite. Sci. Rep..

[23] Pacella A., Andreozzi G.B., Corazzari I., Tomatis M., Turci F. (2018). Surface Reactivity of Amphibole Asbestos. A Comparison between Two Tremolite Samples with Different Surface Area. Period. Miner..

[24] Pacella A., Tomatis M., Viti C., Bloise A., Arrizza L., Ballirano P., Turci F. (2020). Thermal Inertization of Amphibole Asbestos Modulates Fe Topochemistry and Surface Reactivity. J. Hazard. Mater..

[25] Pacella A., Ballirano P., Fantauzzi M., Rossi A., Nardi E., Capitani G., Arrizza L., Montereali M.R. (2021). Surface and Bulk Modifications of Amphibole Asbestos in Mimicked Gamble’s Solution at Acidic PH. Sci. Rep..

[26] Ballirano P., Skogby H., Gianchiglia F., Di Carlo M.C., Campopiano A., Cannizzaro A., Olori A., Pacella A. (2022). Chemical and Structural Characterization of UICC Amosite Fibres from Penge Mine (South Africa). Period. Miner..

[27] Cheary R.W., Coelho A. (1992). A Fundamental Parameters Approach to X-Ray Line-Profile Fitting. J. Appl. Crystallogr..

[28] Young R.A., Young R.A. (1993). Introduction to the Rietveld Method. The Rietveld Method.

[29] Järvinen M. (1993). Application of Symmetrized Harmonics Expansion to Correction of the Preferred Orientation Effect. J. Appl. Crystallogr..

[30] Sabine T.M., Hunter B.A., Sabine W.R., Ball C.J. (1998). Analytical expressions for the transmission factor and peak shift in absorbing cylindrical specimens. J. Appl. Crystallogr..

[31] Ballirano P. (2003). Effects of the Choice of Different Ionization Level for Scattering Curves and Correction for Small Preferred Orientation in Rietveld Refinement: The MgAl_2_O_4_ Test Case. J. Appl. Crystallogr..

[32] (2010). Surface Chemical Analysis—X-ray Photoelectron Spectrometers—Calibration of Energy Scales.

[33] Fantauzzi M., Pacella A., Atzei D., Gianfagna A., Andreozzi G.B., Rossi A. (2010). Combined Use of X-Ray Photoelectron and Mössbauer Spectroscopic Techniques in the Analytical Characterization of Iron Oxidation State in Amphibole Asbestos. Anal. Bioanal. Chem..

[34] Scofield J.H. (1976). Hartree-Slater Subshell Photoionization Cross-Sections at 1254 and 1487 EV. J. Electron Spectros. Relat. Phenomena.

[35] Reilman R.F., Msezane A., Manson S.T. (1976). Relative Intensities in Photoelectron Spectroscopy of Atoms and Molecules. J. Electron Spectros. Relat. Phenomena.

[36] Hirschmann M., Evans B.W., Yang H. (1994). Composition and Temperature Dependence of Fe-Mg Ordering in Cummingtonite-Grunerite as Determined by X-Ray Diffraction. Amer. Miner..

[37] Schott J., Berner R.A., Sjöberg E.L. (1981). Mechanism of Pyroxene and Amphibole Weathering—I. Experimental Studies of Iron-Free Minerals. Geochim. Cosmochim. Acta.

[38] Brantley S.L., Chen Y. (1995). Chemical Weathering Rates of Pyroxenes and Amphiboles. Rev. Mineral. Geochem..

[39] Germine M., Puffer J.H. (2015). Analytical Transmission Electron Microscopy of Amphibole Fibers From the Lungs of Quebec Miners. Arch. Environ. Occup. Health.

[40] Germine M., Puffer J.H. (2020). Tremolite–actinolite fiber coatings of sub-nanometer silica-rich particles in lungs from deceased Quebec miners. Toxicol. Ind. Health..

[41] Schott J., Berner R.A. (1983). X-Ray Photoelectron Studies of the Mechanism of Iron Silicate Dissolution during Weathering. Geochim. Cosmochim. Acta.

[42] Siever R., Woodford N. (1979). Dissolution Kinetics and the Weathering of Mafic Minerals. Geochim. Cosmochim. Acta.

[43] Speil S., Leineweber J.P. (1969). Asbestos Minerals in Modern Technology. Environ. Res..

[44] Hesterberg T.W., Miiller W.C., Musselman R.P., Kamstrup O., Hamilton R.D., Thevenaz P. (1996). Biopersistence of Man-Made Vitreous Fibers and Crocidolite Asbestos in the Rat Lung Following Inhalation. Fundam. Appl. Toxicol..

[45] Hesterberg T.W., Hart G.A., Chevalier J., Miiller W.C., Hamilton R.D., Bauer J., Thevenaz P. (1998). The Importance of Fiber Biopersistence and Lung Dose in Determining the Chronic Inhalation Effects of X607, RCF1, and Chrysotile Asbestos in Rats. Toxicol. Appl. Pharmacol..

[46] Hesterberg T.W., Chase G., Axten C., Miller W.C., Musselman R.P., Kamstrup O., Hadley J., Morscheidt C., Bernstein D.M., Thevenaz P. (1998). Biopersistence of Synthetic Vitreous Fibers and Amosite Asbestos in the Rat Lung Following Inhalation. Toxicol. Appl. Pharmacol..

[47] Bernstein D.M., Chevalier J., Smith P. (2003). Comparison of Calidria Chrysotile Asbestos to Pure Tremolite: Inhalation Biopersistence and Histopathology Following Short-Term Exposure. Inhal. Toxicol..

[48] Enrico Favero-Longo S., Turci F., Tomatis M., Compagnoni R., Piervittori R., Fubini B. (2009). The Effect of Weathering on Ecopersistence, Reactivity, and Potential Toxicity of Naturally Occurring Asbestos and Asbestiform Minerals. J. Toxicol. Environ. Health Part A.

[49] Hawthorne F.C., Oberti R., Hawthorne F.C., Oberti R., Della Ventura G., Mottana A. (2007). Amphiboles: Crystal Chemistry. Amphiboles: Crystal Chemistry, Occurrence, and Health Issues.

