# Supplementary material for: Dissolution Reaction and Surface Modification of UICC Amosite in Mimicked Gamble’s Solution: A Step towards Filling the Gap between Asbestos Toxicity and Its Crystal Chemical Features
Source: Nanomaterials (Basel). 2023 Nov 12;13(22):2933. doi: 10.3390/nano13222933 (PMC10674585; doi:10.3390/nano13222933)
Supplement: Supplementary file 1 [file nanomaterials-13-02933-s001.zip › nanomaterials-2671598-supplementary.pdf]

DISSOLUTION REACTION AND SURFACE MODIFICATION OF UICC AMOSITE IN MIMICKED GAMBLE'S SOLUTION: A STEP TOWARD FILLING THE GAP BETWEEN ASBESTOS TOXICITY AND ITS CRYSTAL CHEMICAL FEATURES

Alessandro Pacella[1,*], Paolo Ballirano[1,2], Maria Cristina Di Carlo[1], Marzia Fantauzzi[3], Antonella Rossi[3], Elisa Nardi[4], Cecilia Viti[5], Lorenzo Arrizza[6], Antonella Campopiano[7], Annapaola Cannizzaro[7], Andrea Bloise[8,*], and Maria Rita Montereali[9]

[1] Department of Earth Sciences, Sapienza University of Rome, Piazzale Aldo Moro 5, I-00185, Rome, Italy

[2] Rectoral Laboratory Fibres and Inorganic Particulate, Sapienza University of Rome, Piazzale Aldo Moro 5, I-00185, Rome, Italy

[3] Department of Chemical and Geological Sciences, INSTM Research Unit, University of Cagliari, I-09042, Monserrato, Cagliari, Italy

[4] Institute for Environmental Protection and Research, ISPRA, via Vitaliano Brancati 48, 00144 Rome, Italy[4]

[5] University of Siena, Department of Physical, Earth and Environmental Sciences, Via Laterina 8, I-53100, Siena, Italy[6]

[6] Microscopy Center, University of L' Aquila, Via Vetoio, Locality Coppito, 67100 L'Aquila, Italy.

[7] National Institute for Insurance against Accidents at Work (INAIL), Department of Medicine, Epidemiology, Occupational and Environmental Hygiene, via Fontana Candida 1, 00078 Monte Porzio Catone, Rome, Italy

[8] Department of Biology, Ecology and Earth Sciences, University of Calabria, V. P. Bucci, I-87036, Arcavacata di Rende, CS, Italy

[9] Italian National Agency for New Technologies, ENEA, Casaccia Research Centre, via Anguillarese 301, I-00123 S. Maria di Galeria, Rome, Italy

* Corresponding author: alessandro.pacella@uniroma1.it; andrea.bloise@unical.it

Table S1. Cell parameters and volume of UICC amosite fibres and agreement factors (as defined in the work of Young, [28]) of the Rietveld refinements. Data of pristine samples taken from Ballirano et al. [26].

	Pristine	A-720h
R_{Bragg} (%)	0.58	0.59
R_{wp} (%)	1.40	1.47
R_p (%)	0.84	1.00
GoF	4.08	4.24
DWd	0.78	0.73
a (Å)	9.55264(17)	9.55226(16)
b (Å)	18.3069(3)	18.3079(3)
c (Å)	5.33487(8)	5.33499(7)
β (°)	101.840(3)	101.841(2)
Vol. (Å^3)	913.11(3)	913.14(3)

Table S2. Relevant bond distances (in Å) of UICC amosite fibres. * Calculated as in table 7 in the work of Hawthorne and Oberti [49]. Data of pristine samples taken from Ballirano et al. [26].

		Pristine	A-720h
T(1)	-O(7)	1.606(5)	1.605(5)
	-O(6)	1.650(10)	1.652(9)
	-O(1)	1.631(12)	1.643(12)
	-O(5)	1.615(12)	1.603(11)
<*T*(1)-O>		1.626	1.626
T(2)	-O(4)	1.609(9)	1.609(9)
	-O(5)	1.615(10)	1.636(10)
	-O(2)	1.621(12)	1.634(11)
	-O(6)	1.659(11)	1.652(11)
<*T*(2)-O>		1.626	1.633
M(1)	-O(3) x2	2.132(9)	2.134(8)
	-O(1) x2	2.079(12)	2.065(12)
	-O(2) x2	2.133(9)	2.136(9)
<*M*(1)-O>		2.115	2.112
<r$_{M(1)}$>		0.755	0.752
M(2)	-O(4) x2	2.064(8)	2.056(8)
	-O(2) x2	2.112(12)	2.102(11)
	-O(1) x2	2.155(9)	2.160(9)
<*M*(2)-O>		2.110	2.106
<r$_{M(2)}$>		0.750	0.746
M(3)	-O(1) x4	2.121(9)	2.122(8)
	-O(3) x2	2.111(15)	2.098(15)
<*M*(3)-O>		2.118	2.114
<r$_{M(3)}$>		0.758	0.754
<<*M*(1,2,3)-O>>		2.114	2.111
<r$_{M(1),(2),(3)}$>		0.754	0.751
M(4)	-O(4) x2	1.997(10)	1.966(10)
	-O(2) x2	2.189(9)	2.179(9)
	-O(6) x2	2.749(10)	2.720(9)
<*M*(4)-O>		2.312	2.288

Table S3. Site scattering (*s.s.*) at A, B and C sites from Rietveld refinement. Data of pristine samples taken from Ballirano et al. [26].

Site	Pristine	A-720h
C		
M(1)	43.5(3)	43.4(3)
M(2)	37.2(2)	37.4(2)
M(3)	22.0(2)	22.5(2)
$\Sigma_{M(1)+M(2)+M(3)}$	102.8(7)	103.3(7)
B		
M(4)	51.0(3)	49.9(3)
A	-	-

Table S4. Site partition at B and C sites from Rietveld refinement. Data of pristine samples taken from Ballirano et al. [26].

	Fe^{2+}/Fe^{3+} partition from <r^M>	
Site	Pristine	A-720h
C		
M(1)	$Mg_{0.60}Fe^{3+}_{0.11}Fe^{2+}_{1.29}$	$Mg_{0.62}Fe^{3+}_{0.15}Fe^{2+}_{1.23}$
M(2)	$Mg_{1.05}Fe^{2+}_{0.95}$	$Mg_{1.04}Fe^{3+}_{0.03}Fe^{2+}_{0.92}$
M(3)	$Mg_{0.29}Fe^{3+}_{0.04}Fe^{2+}_{0.67}$	$Mg_{0.25}Fe^{3+}_{0.09}Fe^{2+}_{0.66}$
$\Sigma_{M(1)+M(2)+M(3)}$	$Mg_{1.94}Fe^{3+}_{0.15}Fe^{2+}_{2.91}$	$Mg_{1.91}Fe^{3+}_{0.27}Fe^{2+}_{2.82}$
B		
M(4)	$Fe^{2+}_{1.59}Mn_{0.29}Ca_{0.12}$	$Fe^{2+}_{1.42}Mn_{0.29}Ca_{0.29}$

Table S5. Results of ICP-OES analyses of the UICC amosite sample after incubation in the mimicked Gamble's solution at pH 4.5 up to 720 hours. Standard deviations (in parentheses) were calculated over three independent measurements.

Sample	Fe (mg/kg)	Ca (mg/kg)	Mg (mg/kg)	Si (mg/kg)
A-1h	720(37)	1435(29)	394(3)	57(1)
A-24h	652(74)	1796(86)	548(23)	403(19)
A-48h	332(295)	2555(637)	615(41)	641(64)
A-168h	459(69)	1852(35)	793(33)	1292(83)
A-720h	not detected	2039(69)	985(36)	2169(62)

Table S6. Binding energy values (eV) of the main photoelectron lines in UICC amosite samples. Average values and standard deviation (in parentheses) over three measurements carried out on different areas of the same sample.

		Binding Energy (eV)				
		**A-1h**	**A-24h**	**A-48-h**	**C-168h**	**A-720h**
**Si $2p_{3/2}$**		102.6 (0.1)	102.7 (0.1)	102.6 (0.1)	102.5 (0.1)	102.5 (0.1)
**O 1s**	O^{2-}	530.4 (0.2)	530.4 (0.2)	530.2 (0.2)	530.2 (0.2)	530.2 (0.2)
	NB-O† and OH^-	531.4 (0.1)	531.3 (0.1)	531.2 (0.1)	531.3 (0.1)	531.2 (0.1)
	B-O‡	532.3 (0.2)	532.3 (0.2)	532.2 (0.2)	532.2 (0.2)	532.2 (0.2)
**Fe $2p_{3/2}$**	Fe (II) - O	709.0 (0.2)	709.0 (0.1)	709.0 (0.1)	709.0 (0.1)	709.1 (0.1)
	Fe (III) - O	710.5 (0.1)	710.6 (0.2)	710.5 (0.1)	710.5 (0.2)	710.6 (0.1)
	FeOOH	711.6 (0.2)	711.6 (0.2)	711.6 (0.2)	711.6 (0.2)	711.6 (0.2)
**Na 1s**		1072.3 (0.1)	1072.4 (0.2)	1072.3 (0.2)	1072.5 (0.2)	1072.4 (0.2)
**Mg 2p**		50.0 (0.2)	50.2 (0.2)	50.2 (0.2)	50.0 (0.1)	50.2 (0.2)

† NB-O: non-bridging oxygen in silicates. ‡ B-O: bridging oxygen in silicates.

Figures

Figure S1. Magnified view (5-80° 2θ) of the Rietveld plots of the refinement of A-720h. Continuous blue line: experimental data; continuous red line: calculated pattern; continuous grey line: difference pattern. Vertical blue lines: single-peak of annite/biotite (ca. 7.25°2θ), stilpnomelane (ca. 8.8° 2θ) and hydrated sulphates (ca. 18 °2θ) (see text for explanation). Vertical short bars: position of calculated Bragg reflections of (from above to below): garnet, quartz, and amosite.

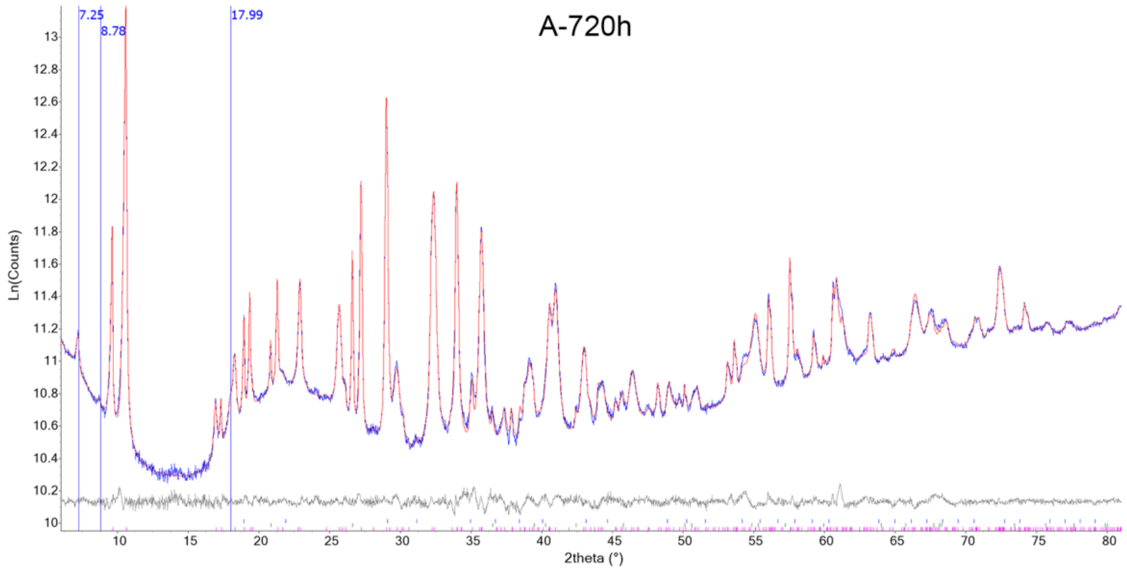

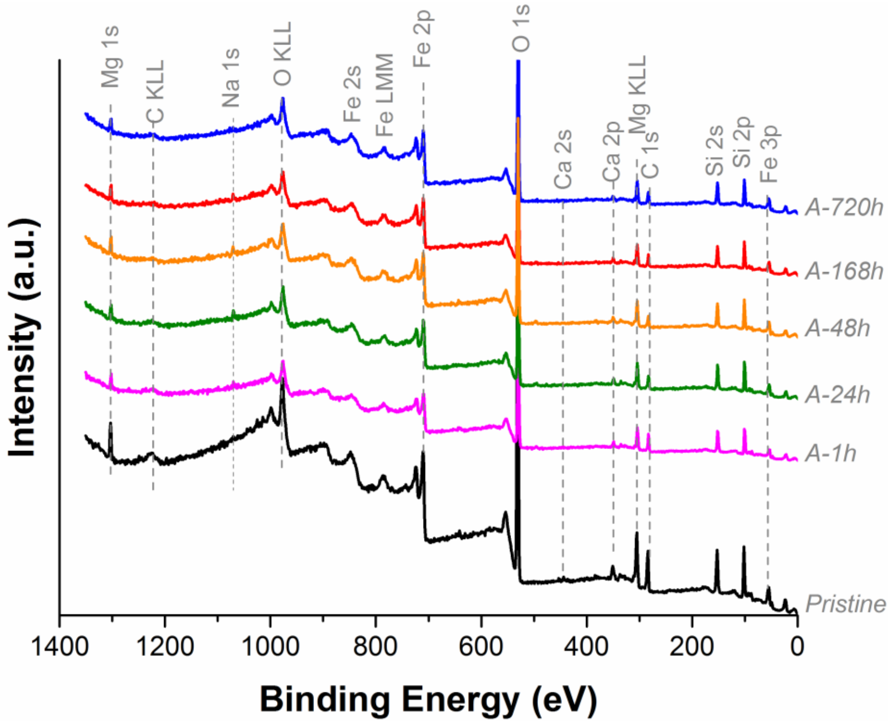

Figure S2. Survey spectra of the investigated UICC amosite samples. X-ray source: Al Kα.

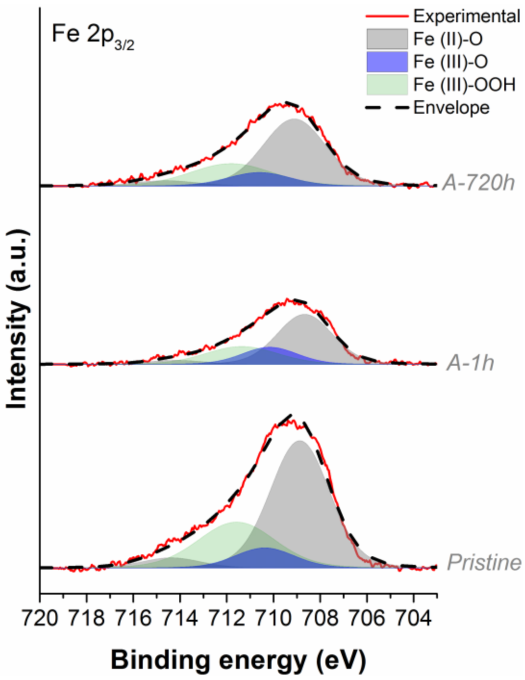

Figure S3. Fe $2p_{3/2}$ peaks of the UICC amosite samples pristine and incubated in mimicked Gamble's solution for 1 hour and 720 hours.
